# Efficacy, Safety and Mechanism of Jinzhen Oral Liquid in the Treatment of Acute Bronchitis in Children: A Randomized, Double-Blind, Multicenter Clinical Trial Protocol

**DOI:** 10.3389/fphar.2022.948236

**Published:** 2022-07-01

**Authors:** Xin Cui, Long Liang, Hongjiao Geng, Yi Liu, Junyu Xi, Junhong Wang, Tee Bee Ching, Eow Gaik Bee, Yan Chai, ShengXian Wu, De Jin, YanMing Xie

**Affiliations:** ^1^ Institute of Basic Research in Clinical Medicine, China Academy of Chinese Medical Sciences, Beijing, China; ^2^ First Affiliated Hospital of Anhui University of Traditional Chinese Medicine, Hefei, China; ^3^ Dongzhimen Hospital Affiliated to Beijing University of Chinese Medicine, Beijing, China; ^4^ AMD Clinic, Johor Bahru, Malaysia; ^5^ Department of Cerebral Neurology, Penang General Hospital, Penang, Malaysia; ^6^ Department of Epidemiology, University of California, Los Angeles, Los Angeles, United States; ^7^ Hangzhou Hospital of Traditional Chinese Medicine, Hangzhou, China

**Keywords:** Jinzhen Oral Liquid (JZOL), acute bronchitis (AB), Ambroxol Hydrochloride and Clenbuterol Hydrochloride Oral Solution (AHCHOS), traditional Chinese medicine (TCM), randomized controlled trial, protocol

## Abstract

**Background:** Acute bronchitis (AB) is a common disease in pediatrics. Prolonged AB may develop into chronic bronchitis. Bronchitis caused by the influenza virus can lead to severe hypoxia or insufficient ventilation, causing great harm to patients and increasing the burden on children and society. Presently, there is no specific treatment for AB except symptomatic supportive treatment. It is urgent to find an effective treatment for AB. Jinzhen Oral Liquid (JZOL) has been found to have a broad spectrum of anti-inflammatory and antiviral effects in previous clinical and basic studies and has a good effect on AB in children. However, the large-sample, randomized, double-blind, head-to-head, evidence-based studies are lacking. The purpose of this protocol is to evaluate the efficacy, safety, and mechanism of JZOL in the treatment of AB in children.

**Methods:** This is a randomized, double-blind, parallel-controlled multi-center clinical trial. The sample size is 500 participants in the intervention group and the control group respectively, with a total of 1000 participants. They will be recruited by 10 hospitals in China. The Intervention group takes JZOL and Ambroxol Hydrochloride and Clenbuterol Hydrochloride Oral Solution (AHCHOS) placebo, while the control group receives AHCHOS and JZOL placebo. The dosage of the two drugs varies according to age and weight. The medication lasts for 7 days. The disappearance time of cough is adopted as the primary outcome. Quality control will be carried out at every stage of data management and processing to ensure that all data are reliable and processed correctly. SAS is used for statistical analysis. Intention-to-treat analysis will be carried out in this trial. All statistical tests are conducted using a two-sided test, and *p* <0.05 would be considered statistically significant.

**Discussion:** We hypothesized that children with AB could get good health benefits from JZOL. This study not only evaluates the clinical efficacy and safety of JZOL but also conducts metagenomics analysis and metabolomics analysis of feces and saliva of participants to study the mechanism of JZOL against AB. Therefore, this protocol evaluates the efficacy, safety, and mechanism of JZOL from a comprehensive perspective, so as to obtain a more solid evidence chain, which will enhance the credibility of the evidence. If successful, this study will provide a high-level evidence-based reference for the treatment of AB in children and future relevant studies.

## Introduction

Acute bronchitis (AB), also known as lobular pneumonia, is a common disease in pediatrics (Koehler, et al., 2019; Wopker, et al., 2020). The main clinical manifestations of the disease are cough, asthma, dyspnea, fever, headache, and general malaise (Kantar, et al., 2017). This disease has complex pathogenic factors and high incidence, accounting for 40% of all healthcare-associated infections in pediatric long-term care facilities (Murray, et al., 2016). Children have lower immunity than adults, so their conditions tend to change quickly. If not treated in time, it may lead to various complications, such as chronic bronchitis, emphysema, chronic obstructive pulmonary disease, *etc*., seriously affecting the quality of life of the children and their guardians (Newcombe, et al., 2010; Rolfsjord, et al., 2016). Moreover, the risk of AB is increased due to complex conditions, frequent device use, immaturity of immunity, and behavioral factors. AB spends 10 times as much in the respiratory virus season as in the non-respiratory virus season (Murray, et al., 2016). AB causes the under-five mortality rate to reach 15 percent, resulting in an estimated 808,694 child deaths (Murray, et al., 2016). Therefore, AB not only has high morbidity and mortality but also imposes a huge economic burden on society and families (Kinkade, et al., 2016; Murray, et al., 2016; Unger, et al., 2017). Currently, there is no specific treatment for AB except symptomatic supportive treatment. Antibiotics are the first choice for pure western medicine (Aabenhus, et al., 2017; Ouldali, et al., 2017). However, in recent years, the abuse of antibiotics has increased the number of drug-resistant bacteria, leading to a prolonged illness, more likely to develop into a severe illness, and then endanger life (Zanasi, et al., 2016; Bauer, et al., 2019; Walters, et al., 2022). Therefore, it is urgent to find an effective treatment for AB.

Traditional Chinese Medicine (TCM) for AB has a long history and has been widely recognized and used in Asian countries (Gottschling, et al., 2013). Jinzhen Oral Liquid (JZOL) is a TCM compound preparation, which originated from the pediatric formula “Antelope Qingfei Prescription,” with a history of more than 400 years (Tao, et al., 2020). JZOL is mainly used for acute bronchitis, mycoplasma pneumonia, viral pneumonia, and other diseases in children (Hu, et al., 2014; Yu, et al., 2019). JZOL consists of eight traditional Chinese herbs including Antelope Horn (*Saigae Tataricae Cornu*), Pingbeimu (*Fritillaria usuriensis Maxim. (Liliaceae))*, Dahuang (*Rheum officinale Baill. (Polygonaceae))*, Huangqin (*Scutellaria baicalensis Georgi (Lamiaceae))*, Niuhuang (*Bos Taurus domesticus Gmelin’s dry gallstones*), Qingmengshi (*Metamorphic biotite schist or chloritic mica carbonate schist*), Shi-gao (*Sulfate minerals gypsum family gypsum*) and Gancao (*Glycyrrhiza glabra L. (Fabaceae))* (Tao, et al., 2020). JZOL has the effect of clearing heat, detoxifying, resolving phlegm, and relieving cough (Tao, et al., 2020). Some researchers used UPLC-Q/TOF-MS technology to analyze the prototype components and metabolites in plasma, urine, bile, and feces of JZOL after intragastric administration in rats. That study described the metabolic profile of JZOL *in vivo* and preliminarily revealed its material basis *in vivo*. And the results showed that the main components absorbed into the blood by JZOL were flavonoids, saponins, and anthraquinones. The main metabolic reactions in rats were hydrolysis, hydroxylation, glycolaldehyde acidification, and sulfation (Zhang, et al., 2021). In addition, a number of clinical studies have also proved the clinical benefits of JZOL in children with refractory mycoplasma pneumoniae pneumonia (RMPP), bronchopneumonia, community-acquired pneumonia, and viral pneumonia (Hu, et al., 2019; Sun, 2020; Yang, et al., 2021; Liu, et al., 2022). In the clinical study of RMPP in children, compared with the control group, JZOL combined with methylprednisolone can effectively improve the clinical symptoms and the level of lung function, and reduce the inflammatory response, with good safety (Liu, et al., 2022). In the clinical study on the treatment of mycoplasma pneumonia in children, JZOL combined with azithromycin can reduce the levels of IL-6, IL-8, and IL-10, regulate the inflammatory response and improve the clinical symptoms with good safety (Sun, 2020).

However, the previous clinical studies lacked large sample, multi-center randomized double-blind controlled studies in terms of trial design. At the same time, in terms of intervention methods, most clinical trials adopted western medicine combined with JZOL compared with western medicine alone, which failed to fully highlight the advantages of JZOL in the treatment of AB. In terms of mechanism research, there are few laboratory studies on the mechanism of TCM treatment of AB. Therefore, a randomized, double-blind, parallel-controlled, multi-center clinical trial is designed to accurately evaluate the clinical efficacy and safety of JZOL in the treatment of AB in children. Meanwhile, this protocol will also explore the *in vivo* mechanism of JZOL in the treatment of AB through metagenomic analysis of stool or blood and metabolomics analysis of stool or blood. If completed, this study will provide high-level evidence-based support and reference for clinical treatment and drug decision-making.

## Materials and Methods

### Study Design

**FIGURE 1:**
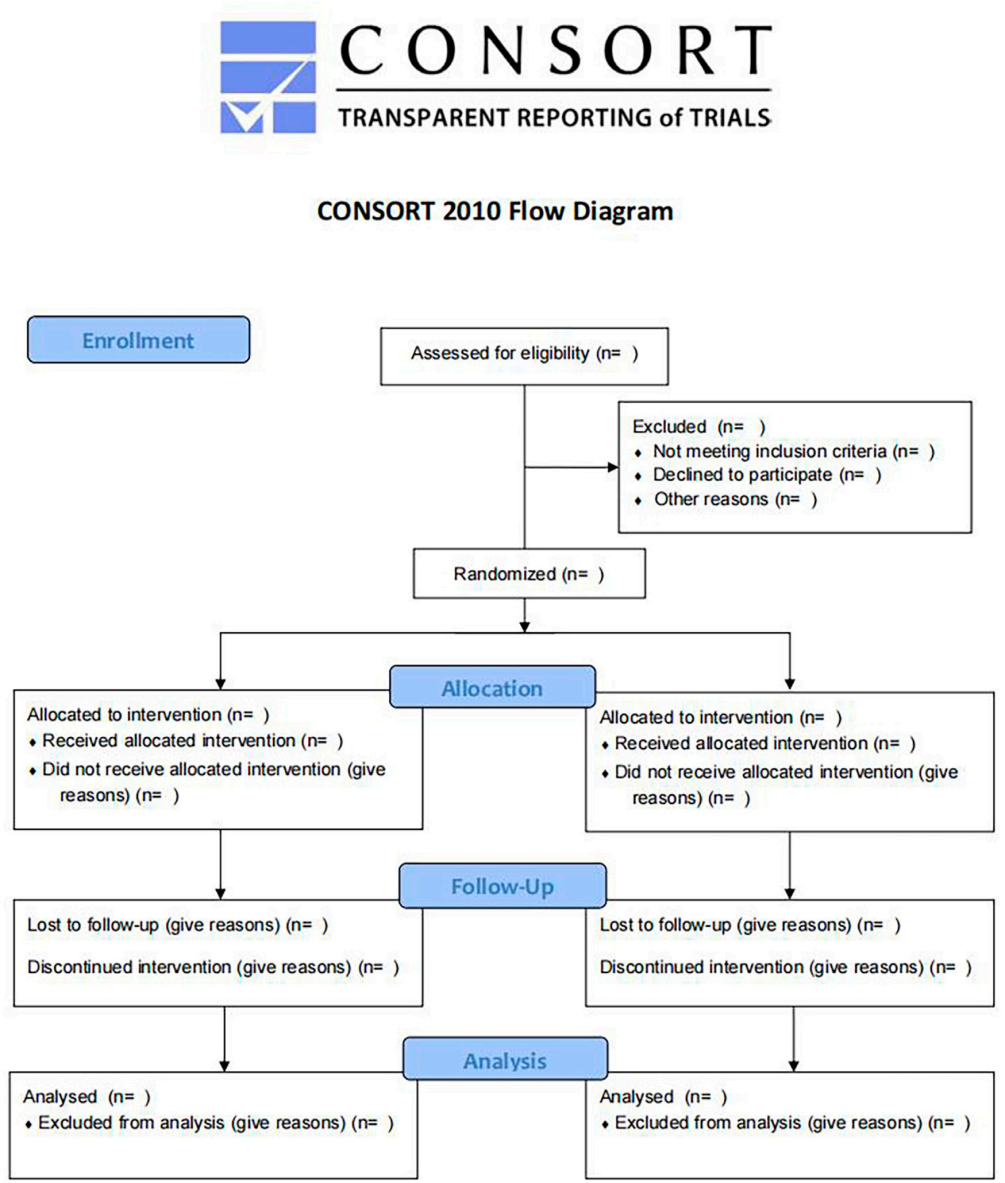
Flow diagram of the trial.

This clinical trial is designed as a stratified, randomized, double-blind, parallel-controlled, multi-center clinical study. The sample size is 500 participants in the intervention group and the control group respectively, with a total of 1000 participants. The trial will be carried out in 10 hospitals in China: *1*) Dongzhimen Hospital Affiliated to Beijing University of Chinese Medicine, *2*) Beijing Children’s Hospital, Capital Medical University, *3*) Children’s Hospital of Soochow University, *4*) Children’s Hospital Affiliated to Shandong University, *5*) The Second Affiliated Hospital of Heilongjiang University of TCM, *6*) Beijing Hepingli Hospital, *7*) Guangzhou Women and Children’s Medical Centar, *8*) Children’s Hospital of Shanghai, *9*) The First Affliated Hospital of Henan University of TCM, *10*) Xuzhou Children’s Hospital. All of these hospitals are qualified and experienced to conduct clinical trials. All enrolled participants will sign an informed consent before randomization. The flow chart of the trial is shown in Figure 1. There are three main objectives of the study. The first aim is to evaluate the effect of JZOL on shortening the course of AB and improving the condition in children. The disappearance time of cough is adopted as the primary outcome. The second aim is to assess the safety of JZOL in the clinical setting. The incidence of adverse events (AEs), routine blood investigations, blood biochemistry investigations, urinalysis, electrocardiogram (ECG), and stool routine tests are the main indicators to evaluate safety. The third aim is to explore the mechanism of action of JZOL in the treatment of AB by feces/saliva metagenomic analysis and feces/blood metabolomics analysis. The protocol will be reported following the Standard Protocol Items for Clinical Trials with Traditional Chinese Medicine 2018: Recommendations, Explanation and Elaboration (SPIRIT-TCM Extension 2018) (Additional file 1) (Dai, et al., 2019).

### Participants

#### Diagnostic Criteria

(1) Diagnostic criteria of Western medicine

AB is diagnosed if the child has respiratory symptoms such as cough, combined with lung crackles and/or chest radiographic changes (Hu, et al., 2014).

AB is diagnosed when there are primary symptoms, plus three secondary symptoms, concerning tongue and pulse condition (China Association of Chinese Medicine, 2012).

#### Inclusion Criteria

(1) Participants with AB under the diagnostic criteria of Western medicine.
(2) Those who meet the TCM diagnostic criteria of phlegm-heat stasis in lungs.
(3) Participants aged 2–14 years.
(4) Cough (day + night) score ≥4 points.
(5) Duration of disease ≤48 h, no antibiotics, antitussives, phlegm-reducing drugs, and other Chinese or Western medicine that influence cough has been used before treatment.
(6) The informed consent process complies with the regulations, and the legal representative and/or the child (≥8 years old) signs the informed consent.

#### Exclusion Criteria

(1) Participants with severe bronchitis, which is difficult to distinguish from pneumonia in the early stage.
(2) Participants with acute infectious diseases such as measles, whooping cough, and influenza.
(3) Participants with single acute upper respiratory tract infection, suppurative tonsillitis, asthmatic bronchitis, bronchial asthma, bronchiolitis pneumonia, tuberculosis, or tumor.
(4) White blood cell count >12.0 × 10^9^/L, or a lot of purulent sputum in the lungs.
(5) Participants with severe malnutrition or immunodeficiency.
(6) Participants with severe diseases involving the heart, liver, kidney, digestive and hematopoietic system.
(7) Allergic to JZOL or Ambroxol Hydrochloride and Clenbuterol Hydrochloride Oral Solution (AHCHOS).
(8) Participants who are taking epinephrine, isoproterenol, or other catecholamines.
(9) Participants who are taking monoamine oxidase inhibitors or tricyclic antidepressants.
(10) Participants who are taking propranolol or other non-selective β-blockers.
(11) Participants who are taking large amounts of other sympathetic stimulants.
(12) Participants who are deemed unsuitable for inclusion in this study by the researcher.

#### Drop-Out Criteria

(1) Participants who develop an allergic reaction or serious adverse event (SAE) would be withdrawn from the trial according to the doctor’s judgment.
(2) Participants with other concomitant diseases occurring during the trial, which affected the assessment of efficacy and safety of JZOL.
(3) Participants whom have poor compliance, changed their medication midway, or added drugs prohibited under this study.
(4) Any reason breaking the double-blind setting of this clinical trial.
(5) After medication, if the total score of cough and expectoration increased by four points or the armpit temperature >38.5°C continued for more than 48 h, or the child developed bronchial pneumonia, the tested medication should be stopped immediately, effective treatment should be given. The child should complete all laboratory tests, be withdrawn from the study, and be considered an invalid case.
(6) Participants who are found to have serious violations of inclusion or exclusion criteria after being enrolled in this study.

#### Voluntary Withdrawal of Participants

(1) The participants and their guardians are unwilling or unable to continue the trial for any reason and request the physician in charge to withdraw the candidate from the trial.
(2) Participants who no longer receive medication and testing, and were lost to follow-up visits.

#### Conditions for Suspension

(1) If an SAE occurs during the study, the trial should stop immediately.
(2) If there is a major error in the trial, or a serious deviation of protocol during implementation, which makes it difficult to evaluate the drug efficacy and safety, the trial should be discontinued.
(3) If the drug has a poor therapeutic effect or no clinical value during the trial, the trial should be stopped.
(4) The sponsor requests to stop the trial.
(5) The administrative authority cancels the trial.

### Sample Size Calculation

In this study, the disappearance time of cough is used as the primary outcome. According to previous studies, the disappearance time of cough in the experimental group and the control group is about 5 ± 2 days. The non-inferiority threshold of this study is set as 0.5 days, unilateral α = 0.025, power = 0.95, and the PASS (V14.0) is selected to estimate the sample size. Based on the calculation, 417 cases are needed for each group. After considering the proportion of withdrawals, the sample size of this study is 500 cases for each group, with a total of 1000 cases.

### Randomization and Blindness

In this protocol, the stratified block randomization method is adopted. Participants will be stratified according to age. The age stratification is divided into five categories, which are 2–3 years old, 4–5 years old, 6–7 years old, 8–12 years old, and more than 12 years old. SAS 9.4 is used to generate random numbers and corresponding treatment groups. Random numbers are assigned by a central random platform (DAS for IWRS), and competitively selected by each clinical subcenter. There are two copies of the random scheme, which were sealed and stored respectively in the sponsor and the designer of the study scheme.

The double-blind method is used in this trial. Participants, clinicians, and outcome evaluators will be blinded. A two-stage blind design also will be used in the study. In this design, the statistical analyst will not know the grouping of medications in the study. Thus, the statistical analyst also will be blinded. To ensure the quality of blinding, all drugs and placebo are required to be uniformly packaged, while guaranteeing that there is no difference between real drugs and placebo in shape, color, sizer, taste, and smell. Entrust a third party to randomly number the corresponding medicines according to the random coding table and paste them in a conspicuous position in the external packaging of the medicines. The researchers logged on to the designed central random platform to obtain the random numbers of participants, and the pharmacy administrators of each clinical unit distributed the corresponding medicines to the patients according to the random number, to ensure that both researchers and patients were blinded. All the outcome measures and statistical analysts were not involved in the implementation of the trial, nor did they know the study design and hypotheses. If the participants experience SAEs during this trial, the principal investigator at each center has the authority to log in to the central randomization platform to unblind the patients, the reason for urgent unblinding should be noted, dated, and recorded on case report form (CRF).

### Intervention Method

The Intervention group takes JZOL and AHCHOS placebo, while the control group receives AHCHOS and JZOL placebo. Both drugs and their placebos are taken orally. According to different ages and weights, the dosages of the two drugs are shown in Table 1. The conditions treated by the two drugs in this study are the same as publicly available information. Ibuprofen Suspension will be taken when two groups of participants appear in an emergency. The medication lasts for 7 days. The treatment medicine and their placebos are provided by Jiangsu Kanion Pharmaceutical Co., Ltd according to the double-blind principle. The schedule of enrollment, interventions, and assessments is shown in Table 2.

**TABLE 1:** Usage instructions for both drugs.

Jinzhen oral liquid and its placebo		
**Age**	**Dosage**	
2–3 years old	10 ml each time, twice a day	
4–7 years old	10 ml each time, three times a day	
8–14 years old	15 ml each time, three times a day	
Ambroxol Hydrochloride and Clenbuterol Hydrochloride Oral Solution and its placebo		
** Age**	**Weight**	**Dosage**
** 2–3 years old**	12–16 kg	7.5 ml each time, twice a day
** 4–5 years old**	16–22 kg	10 ml each time, twice a day
** 6–12 years old**	22–35 kg	15 ml each time, twice a day
** More than 12 years old**	—	20 ml each time, twice a day

Note: In case of inconsistency between age and weight, the dosage can be adjusted according to their weight.

**TABLE 2:** Flow chart for clinical trial.

Stage procedure	Screening stage /Baseline (1st Visit)	During treatment (2nd Visit)<	After treatment (3rd Visit)
	−1 ∼ 0 days	5 ± 1 day	7 ± 1 day
Patient screening	×	—	—
Sign informed consent form	×	—	—
Fill in demographic information	×	—	—
Past medical history	×	—	—
Medical comorbidities and current medication	×	—	—
Chest radiograph	×	—	—
General examination	×	×	×
Physical examination	×	×	×
TCM syndrome scoring and grading	×	×	×
Routine blood investigations, urinalysis and stool examination	×	—	×
Liver function tests (ALT, ALP, AST, TBIl, γ-GT) and kidney function tests (BUN, Cr)	×	—	×
ECG	×	—	×
Stool/salivary metagenomic analysis	×	×	×
Stool/blood metabolomics analysis	×	×	×
Distribution and withdrawal of the remaining intervention medication	×	—	×
Distribution and withdrawal of patients’ medication diary record cards	×	×	×
Adverse events record	—	×	×
Combination therapy medication record	—	×	×
Conclusion (Trial Summary)	—	—	×

#### Guidelines for Drug Combination

(1) During the trial, expectorants, bronchodilators, and other Chinese and Western medicines with antitussive and expectorant effects are not allowed to be used. For example, salbutamol, ipratropium bromide, budesonide, acetylcysteine, terbutaline, ambroxol hydrochloride, which are western medicines commonly used for expectoration and bronchiectasis; and compound fresh bamboo juice, Feilike Mixture, cough syrup, which are Chinese patent medicine used to remove phlegm and relieve cough.
(2) Antibiotics should not be given routinely. Whenever needed, antibiotics can be used under the doctor’s discretion, but the duration, dosage, and frequency should be recorded in detail.
(3) When the axillary temperature exceeds 38.5°C during the study, physical cooling or antipyretic analgesics (e.g. ibuprofen suspension) can be used together, and usage information should also be recorded in detail.
(4) The physician should ask the participants to bring all medication taken by themselves during the follow-up to check for combination medication. For the drugs or other treatments that must be taken continuously due to the combination of diseases, the name, duration, dosage, and frequency of the drugs must be recorded in the medical record, so as to facilitate the summary, analysis and reporting later.

#### Outcome Evaluation

##### Primary Outcome

The disappearance time of cough should be recorded daily, then evaluated at the study endpoint.

##### Secondary Outcome

(1) Area Under Curve (AUC) of cough and expectoration symptom score-time, cough symptom score (Table 3), antitussive onset time, clinical recovery time, and TCM syndrome score (Li, 2015) (Table 4) will be daily recorded and evaluated at the study endpoint.
  i. Cough disappeared: cough symptom score (daytime + night) ≤1 point, and maintained for 24 h or more.
  ii. Antitussive onset time: the number of days required for cough symptom score to decrease by one point after taking the medicine.
  iii. Clinical recovery: cough, expectoration and pulmonary symptoms are all mild after treatment, without affecting study, living and sleep.
  iv. Evaluation criteria for the efficacy of TCM syndromes
    A. Clinical recovery: the total score decreased by ≥90%.
    B. Significant effect: the total score decreased by ≥70% and <90%.
    C. Effective: the total score decreased by ≥30% and <70%.
    D. Ineffective: those who fail to meet the above standards.
(2) The disappearance rate of cough, sputum and pulmonary rales will be evaluated at the study endpoint.
(3) The usage of antibiotics, antipyretics or analgesics will be evaluated at the study endpoint.

**TABLE 3:** Cough symptom scoring.

Score	Daytime symptom	Night symptom
0	No cough	No cough
1	Short occasional cough	Short occasional cough during sleep
2	Frequent cough, daily activities mildly affected	Slight disturbance of sleep due to cough
3	Frequent cough, daily activities severely affected	Severe disturbance of sleep due to cough

**TABLE 4:** Quantitative Grading evaluation for TCM syndrome.

Grading symptoms		(−)	(+)	(++)	(+++)
Primary symptoms		0	1	2	3
Cough	Daytime	No cough	Short occasional cough	Frequent cough, daily activities mildly affected	Frequent cough, daily activities severely affected
	Night	No cough	Short occasional cough during sleep	Slight disturbance of sleep due to cough	Severe disturbance of sleep due to cough
Expectoration	Daytime	No expectoration	Phlegm is whitish and slightly sticky	Phlegm is whitish/yellowish and sticky	Phlegm is yellowish and sticky
	Night	No expectoration	Phlegm is easy to cough out	Phlegm is slightly difficult to cough out	Phlegm is difficult to cough out
Secondary Symptoms		0	1	2	3
Fever (24 h maximum axillary temperature)		≤37.2°C	37.3–37.9°C	38.0–38.5°C	>38.5°C
Thirsty		No	Yes	Yes	Yes
Facial Erythema		No	Yes	Yes	Yes
Dysphoria		No	Yes	Yes	Yes
Scanty dark-colored urine		No	Yes	Yes	Yes
Dry stool		No	Yes	Yes	Yes
Tongue and pulse condition		Normal	Abnormal	Others	
Tongue		Pinkish	Reddish	—	
Tongue coating		Thin whitish coating	Yellowish coating	—	
Pulse condition		Normal	Slippery and rapid	—	

Note : Children who cannot expectorate is not included.

### Safety Evaluation

General physical examination, such as body temperature, pulse rate, respiration and blood pressure, are measured at baseline, during the treatment period and at the end of the treatment. Routine blood investigations, urinalysis, stool routine tests, liver function tests, renal function tests and ECG are examined at baseline and at the end of the trial. Participants who are normal before treatment but abnormal after treatment should be re-examined regularly until the end of follow-up. Possible AEs and allergic reactions will be observed at any time after medication. If an AE occurs during the trial, the sponsor shall be responsible for the cost of treatment until the AE disappears or the condition becomes stable.

### Mechanism Investigation

In order to understand the mechanism of the action for efficacy of JZOL in the treatment of AB, stool, saliva and blood are collected three times, i.e., before treatment, 5 days after treatment and at the end of treatment, followed by stool/saliva metagenomics analysis and stool/blood metabolomics analysis. In order to exclude the effect of antibiotics on the intestinal flora and its metabolites, participants who received antibiotics during treatment should be excluded.

### Quality Control

In order to ensure the quality of the trial and the implementation of quality assurance system, investigators are required to carry out clinical trials according to standard operating procedures (SOPs). All the results and findings in the trial should be verified to ensure the reliability of the data and to ensure that the conclusions of clinical trials are derived from the original data. Researchers must carry out quality control at every stage of data processing to ensure that all data are reliable and processed correctly.

This trial will carry out quality control in the following aspects: progress of the trial; qualification of clinical sub-center; qualification of researchers; mastery of procedures; scientificity, authenticity, accuracy, and completeness of CRF; preservation of files; program implementation; AEs; preservation and storage of drugs; collection and management of biological samples; written informed consent; patients compliance; and laboratory examination data. In particular, the authenticity and accuracy of the CRF, the implementation of the program, and the determination of AEs will be strictly inspected. A diary record card for this trial will be provided to record medication status, symptom and syndrome score. In this way, recall bias can be avoided and study quality and compliance can be improved.

Before the drafting of the protocol, the study will invite experts in the pediatric and respiratory fields to hold a design meeting to determine the test objectives, ideas, and methods. After the protocol is drafted, all study sub-centers will be convened to discuss specific operation details and enhance the operability of the protocol. After the protocol is drafted, all study sub-centers will be convened to discuss specific operation details and enhance the operability of the protocol. During the implementation of the trial, research training sessions will be held in each clinical sub-center to strengthen the researchers’ and quality control staff’s grasp and understanding of the trial scheme and its key procedures. At the same time, interim coordination meetings will be convened as appropriate to discuss and solve technical problems in the operation process. After the completion of the trial, all clinical sub-centers will be invited to participate in the trial summary meeting to discuss the Multi-center Summary Report.

### Data Management

Electronic data management and direct-attached storage for Electronic Data Capture (EDC) system are used in this study. The Data Management Plan (DMP) is written by the data manager as the guidance for data management and is approved by the sponsor. The data management will be carried out in accordance with the time, content, and method defined by the DMP. The integrity of the data will be checked by reviewing whether all paper and electronic materials are filled in and archived following SOPs. The authenticity of participants will be checked by telephone follow-up. Paper materials of the data will be collected after passing the review by composition of data monitoring committee. Researchers responsible for data input will log in to the established EDC and enter data into eCRF in the principle of double-person and double-enter, and conduct consistency tests to ensure the accuracy of the data.

### Statistical Analysis

SAS will be used for statistical analysis. Intention-to-treat analysis will be carried out in this trial, which refers to the analysis of all cases that have been randomized and used drugs at least once. All statistical tests are conducted using a two-sided test, and *p* <0.05 would be considered statistically significant. The missing data will be filled up by carrying forward the last measured value in this trial. Covariance analysis is used for the evaluation of the cough disappearance time. The group is taken as a fixed effect, and the baseline value of the cough symptom score is taken as covariance into the model for analysis. The difference between the different values of the two groups and their bilateral 95% confidence intervals are calculated. Then, the lower limit of bilateral 95% confidence intervals and the preset non-inferiority standard are compared to determine whether the test group is inferior to the control group. In addition, the subgroup analysis is performed according to age (2–3 years, 4–7 years, and 8–14 years). Measurement methods and data sets of secondary outcomes are shown in Table 5. In terms of safety, the number of AEs and SAEs during the study will be listed, and their incidence will be calculated.

**TABLE 5:** Secondary outcome.

Secondary outcome	Test method	Data set
Time-lapse analysis of measured value and changed value of cough symptom score	T test is used to compare the differences between groups	FAS^a^
AUC of cough and expectoration symptom score - time		
Onset of cough relief		
Clinical recovery time		
Disappearance rate of cough, expectoration and lung rales	χ2 test or Fisher’s exact probability method is used to compare the differences between groups	
Use of antibiotics, antipyretics and analgesics		
Analysis on the curative effect of TCM syndrome	CMH chi-square is used to compare the differences between groups	

a FAS, Full Analysis Set; AUC, Area Under Curve.

## Discussion

Prolonged AB may develop into chronic bronchitis (Kinkade, et al., 2016). Bronchitis caused by the influenza virus can lead to severe hypoxia or insufficient ventilation, causing great harm to patients and increasing the burden on children and society (Unger, et al., 2017). Ciliated epithelial cells will be damaged when airway epithelial tissues are affected by air pollutants, viruses, bacteria, endotoxins, and other pathogenic factors. At the same time, this process will be accompanied by the activation of the corresponding cell membrane G protein receptor, which will activate the intracellular inflammatory stress response and start the downstream inflammatory response pathway, resulting in acute respiratory epithelial injury (Mokra, et al., 2015; Deshpande, et al., 2020). When an acute respiratory epithelial injury occurs, neutrophil infiltration may occur in the respiratory tract, releasing a large number of inflammatory factors, oxygen free radicals, proteolytic enzymes and so on (Narasaraju, et al., 2011). Under this action, the epithelium of the respiratory tract can undergo the reaction of cell self-melting, cilia necrosis and abscission (Narasaraju, et al., 2011). In the treatment of AB, antibiotics are the first choice for Western medicine intervention. However, the abuse of antibiotics has led to an increasing number of clinical drug resistance events. Some studies have shown that the resistance of Spn to β -lactam antibiotics in hospitalized children has decreased, but the resistance to erythromycin and other commonly used antibacterial drugs is still severe, and there are a large number of multiple drug resistance (Lin, et al., 2022). Therefore, there is an urgent need for a safe and effective strategy to treat AB.

JZOL is a compound prescription of TCM (Tao, et al., 2020). It can effectively improve lipopolysaccharide (LPS)-induced interstitial edema of lung tissue. JZOL can also reduce the levels of inflammatory cytokines, tumor necrosis factor -α (TNF-α), and interleukin (IL)-1β (Zong, et al., 2018). Besides, it can inhibit or kill the influenza virus, Coxsackie virus, respiratory syncytial virus, and mycoplasma pneumonia (Xiao, et al., 2008; Hou, et al., 2009; Xiao, et al., 2009). Experimental studies *in vitro* and *in vivo* have shown that Dahuang can inhibit a variety of viruses by inhibiting the viral synthesis and reducing the number of virus replication (Lin, et al., 2016). Dahuang also exerts strong anti-inflammatory effects by inhibiting the production of pro-inflammatory factors such as IL-6, IL-1β, and TNF-α and inhibiting the NF-κB inflammatory signaling pathway (Ge, et al., 2017). An experiment showed that Gancao could inhibit the proliferation of HIV-1, SARS, and other viruses, which indicated that Gancao had good antiviral activity (Yi, et al., 2021). Gancao can also regulate the number and function of lymphocytes, and inhibit the levels of inflammatory mediators and proinflammatory cytokines, indicating that glycyrrhiza has a good anti-inflammatory effect (Li et al., 2021). Animal experiments showed that peimisine had a good therapeutic effect on LPS-induced airway mucus hypersecretion model and acute lung injury mice (Cui, et al., 2015; Wang, et al., 2016). Peiminine can treat bleomycin-induced lung injury by reducing TGF-β/MAPK ERK and MEK1/2 cell signal transduction (Guo, et al., 2016a; Guo, et al., 2016b). These *in vitro* and *in vivo* studies have proved that JZOL has broad spectrum antiviral and anti-inflammatory effects, and has a potential therapeutic effect on AB.

Some clinical studies have come to the same conclusion. In a clinical study of AB in children, JZOL combined with atomized inhalation of budesonide in the treatment of AB achieved a higher degree of clinical efficacy satisfaction, which can effectively shorten the rehabilitation time of children with a low incidence of AEs (Weng, et al., 2021). Meanwhile, another clinical study has shown that, compared with the control group, JZOL combined with ambroxol and clenbuterol tablets can significantly relieve the clinical symptoms of cough, wheezing and other symptoms in children with AB, and improve the level of inflammation with fast onset of effect (Li, 2021). These studies have demonstrated clinical efficacy and safety of JZOL. However, there is still a lack of large-sample, multi-center randomized double-blind controlled studies comparing JZOL head-to-head with Western medicine and the mechanism of its effect *in vivo*.

Even if the use of Chinese compound prescription is on the rise in China (Jin, 2021), the number of high-level evidence-based clinical trials is limited. To date, this study is the first prospective, multi-center, randomized controlled trial of JZOL in the treatment of AB in children strictly according to CONSORT Extension for CHM Formulas (Consort-CHM Formulas 2017) (Cheng, et al., 2017). Simultaneously, we selected ten hospitals from different regions of China as sub-centers of this clinical study, which can reduce regional bias and obtain more accurate results. In addition, standardized methods such as stratified block randomization, double blindness, and double simulation were adopted in this study, which played a positive role in further promoting and improving the level of the evidence-based methodology of TCM worldwide. Furthermore, this study not only evaluates the clinical efficacy of JZOL but also conducts metagenomics analysis and metabolomics analysis of feces and saliva of participants to study the mechanism of JZOL against AB. Therefore, this protocol evaluates the efficacy, safety, and mechanism of JZOL from a comprehensive perspective, so as to obtain a more solid evidence chain, which will enhance the credibility of evidence.

Although strict quality control will be carried out in this study, there might also be potential limitations for this study. Firstly, different hospitals use different testing machines and testing standards, which may lead to test data bias. Secondly, the efficacy criteria of TCM syndromes are not included in the international guidelines. However, the evaluation criteria of TCM efficacy in this study were determined after several rounds of discussion by high-level TCM experts and have been highly recognized in the TCM guidelines (Li, 2015).

We hypothesized that children with AB could get good health benefits from JZOL. If successful, this study will provide a high-level evidence-based reference for the treatment of AB in children and future relevant studies.

## Data Availability

The raw data supporting the conclusions of this article will be made available by the authors, without undue reservation.

## Abbreviations

AB, acute bronchitis; AEs, adverse events; AHCHOS, ambroxol hydrochloride and clenbuterol hydrochloride oral solution; AUC, area under curve; CRF, case report form; DMP, data management plan; ECG, electrocardiogram; EDC, electronic data capture; IL-1β, interleukin-1β; JZOL, Jinzhen Oral Liquid; LPS, lipopolysaccharide; RMPP, refractory mycoplasma pneumoniae pneumonia; SAE, serious adverse event; SOPs, standard operating procedures; TCM, traditional Chinese medicine; TNF-α, tumor necrosis factor -α.
