# Supplementary material for: Efficacy, Safety and Mechanism of Jinzhen Oral Liquid in the Treatment of Acute Bronchitis in Children: A Randomized, Double-Blind, Multicenter Clinical Trial Protocol
Source: Front Pharmacol. 2022 Jul 1;13:948236. doi: 10.3389/fphar.2022.948236 (PMC9283571; doi:10.3389/fphar.2022.948236)
Supplement: Supplementary file 1 [file Table1.DOCX]

**Ethics Committee of Dongzhimen Hospital Affiliated to Beijing University of Chinese Medicine**

**Approval Notice Template**

Project No.: DZMEC-KY-2019-183-01

Project Name: A randomized, double-blind, parallel-controlled, multi-center clinical trial will be conducted to evaluate the efficacy, safety and mechanism of Jinzhen Oral Liquid in the treatment of acute bronchitis in children.

|  | |
| --- | --- |
| Principal Investigator: Wu Shengxian | Project category: Scientific research |
| Applicant: Jiangsu Kanion Pharmaceutical Co., Ltd. | Contract Research Organization (CRO): Not applicable |
| Review category: Amendment review | Review method: ☑ quick review □ meeting review. |
| Examiners: Wang Lan, Xue Chunmiao | Date of review: July 3, 2020 |

Approval document (indicating version number and date):

1. Revised clinical research protocol (Version number: V1.2; Version date: May 29th, 2020)

2. Amended informed consent form (Version number: V2.1; Version date: May 29th, 2020)

3. Case report form (Version number: V1.1; Version date: May 29th, 2020)

Review basis: According to the NMPA " Good Clinical Practice (GCP) of Pharmaceutical Products" (2020) and "Medical Device Clinical Trial Quality Management Practice" (2016), Helsinki Declaration of World Medical Association (2013), the International Ethical Guidelines for Human Biomedical Research of the International Medical Science Organization Committee (2016), and the Measures for Ethical Review of Biomedical Research Involving Peopleof the National Health and Family Planning Commission (No.11, 2016) , etc.

Review decision: Agreed.

Note:

1. Researchers must strictly use the informed consent form and research plan approved by the examination.

2. Before the start of the research, please complete the clinical trial registration.

3. Any research project that involves Chinese human genetic resources and needs to be submitted for approval should be informed that it can only be started after obtaining the approval of China Human Genetic Resources Management Office.

4. If the main researcher is changed during the research process, and any changes to the clinical research plan, informed consent form, recruitment materials, etc. are made, please submit the application for amendment review.

5. In case of serious adverse events, please submit the serious adverse events report in time.

6. Please submit the research progress report one month before the deadline according to the annual/regular follow-up frequency stipulated by the ethics committee.

7. If the study includes participants who do not meet the inclusion criteria, meet the exclusion criteria, or meet the suspension criteria without withdrawing from the study; or the wrong treatment or dose was given, the combination of drugs prohibited by the protocol was given, or the study was not carried out in accordance with the protocol; or it may have adverse effects on the interests and health of the participants or other situations that violate the GCP, the sponsor/supervisor/researcher shall submit a violation plan report.

8. If the applicant suspends or prematurely terminates the clinical study, please submit the suspension/termination report in a timely manner.

9. After completing the clinical trial, please submit the final report.

Time: July 3, 2020

Venue: Dongzhimen Hospital Affiliated to Beijing University of Chinese Medicine

Contact: Han Xueting 010-84012772
