# Supplementary material for: Efficacy, Safety and Mechanism of Jinzhen Oral Liquid in the Treatment of Acute Bronchitis in Children: A Randomized, Double-Blind, Multicenter Clinical Trial Protocol
Source: Front Pharmacol. 2022 Jul 1;13:948236. doi: 10.3389/fphar.2022.948236 (PMC9283571; doi:10.3389/fphar.2022.948236)
Supplement: Supplementary file 2 [file Table2.DOCX]

**Additional file 1** **SPIRIT-TCM** **Extension** **2018** **checklist**

| Item | *SPIRIT* *2013* *statement* | SPIRIT-TCM Extension 2018 | Addressed on page number |
| --- | --- | --- | --- |
| ***Administrative*** ***information*** | | |  |
| Title | *1.* *Descriptive* *title* *identifying* *the* *study* *design,* *population,* *interventions,* *and,* *if* *applicable,* *trial* *acronym* | 1a. Specify the patient population in terms of 1) a WM-defined disease, 2) a WM-defined  disease with a specific TCM *Pattern,* or 3) a TCM *Pattern.* | 1 |
|  |  | 1b. Specify the intervention, in terms of 1) CHM formula, 2) acupuncture, 3) moxibustion,  or 4) other TCM therapy(ies). | 1 |
| Trial  registration | *2a.* *Trial* *identifier* *and* *registry* *name.* *If* *not* *yet* *registered,* *name* *of* *intended* *registry* |  | 2 |
|  | *2b.* *All* *itemsfrom* *the* *World* *Health* *Organization* *Trial* *Registration* *Data* *Set* |  | NA |
| Protocol  version | *3.* *Date* *and* *version* *identifier* |  | NA |
| Funding | *4.* *Sources* *and* *types* *offinancial,* *material,* *and* *other* *support* |  | 12 |
| Roles and responsibilities | *5a.* *Names,* *affiliations,* *and* *roles* *of* *protocol* *contributors* |  | 1,12 |
|  | *5b.* *Name* *and* *contact* *informationfor* *the* *trial* *sponsor* |  | 1 |
|  | *5c.* *Role* *of* *study* *sponsor* *andfunders,* *if* *any,* *in* *study* *design; collection,* *management,* *analysis,* *and* *interpretation* *of* *data;* *writing* *of* *the* *report;* *and* *the* *decision* *to* *submit* *the* *reportfor* *publication,* *including* *whether* *they* *will* *have* *ultimate* *authority* *over* *any* *of* *these* *activities* |  | 12 |

|  | *5d.* *Composition,* *roles,* *and* *responsibilities* *of* *the* *coordinating* *centre,* *steering* *committee,* *endpoint* *adjudication* *committee,* *data* *management* *team,* *and* *other* *individuals* *or* *groups* *overseeing* *the* *trial,* *if* *applicable* *(see* *Item* *21afor* *data* *monitoring* *committee)* |  | 9 |
| --- | --- | --- | --- |
| ***Introduction*** | | |  |
| Background  and rationale | *6a.* *Description* *of* *research* *question* *and justification for undertaking* *the* *trial,* *including* *summary* *of* *relevant* *studies* *(published* *and* *unpublished)* *examining* *benefits* *and* *harms for* *each* *intervention* | 6a. 1 Provide the background and rationale of the research question with TCM theory. | 2-3 |
|  |  | 6a.2 Describe the rationale of the utilized TCM interventions with references. | 2-3 |
|  |  | 6a.3 Provide the rationale of adding experimental TCM interventions if WM intervention is used as basic or combined remedy. If possible, the potential interaction between WM intervention and TCM intervention (especially for CHM) should also be explained with  related reference(s). | NA |
|  | *6b.* *Explanationfor* *choice* *of* *comparators* | 6b. Describe the rationale and principle(s) for selecting comparators corresponding to certain interventions (i.e. CHM formula, acupuncture, moxibustion or other TCM  interventions), considering 1) comparable with tested intervention; 2) success of blinding. | 3 |
| Objectives | *7.* *Specific* *objectives* *or* *hypotheses* | 7. State the objectives or hypotheses regarding the specific TCM intervention for 1) a WM- defined disease, 2) a WM-defined disease with a specific TCM *Pattern* or 3) a TCM  *Pattern*. | 3-4 |
| Trial design | *8.* *Description* *of* *trial* *design* *including* *type* *of* *trial* *(eg,* *parallel* *group,* *crossover,factorial,* *single* *group),allocation* *ratio,* *and framework* *(e.g,* *superiority,equivalence,non inferiority, exploratory)* |  | 3-4 |
| ***Methods*** | | |  |
| ***Participants,*** ***interventions,*** ***and*** ***outcomes*** | | |  |

| Study setting | *9.* *Description* *of* *study* *settings* *(eg,* *community* *clinic, academic* *hospital)* *and* *list* *of* *countries* *where* *data* *will* *be* *collected.* *Reference* *to* *where* *list* *of* *study* *sites* *can* *be* *obtained* |  | 3-4 |
| --- | --- | --- | --- |
| Eligibility  criteria | *10.* *Inclusion* *and* *exclusion* *criteria for* *participants.* *If applicable,* *eligibility* *criteria for* *study* *centres* *and* *individuals* *who* *will* *perform* *the* *interventions* *(e.g, surgeons, psychotherapists)* | 10a. State whether participants with a specific TCM *Pattern* will be recruited, in terms of 1) diagnostic criteria, and 2) inclusion and exclusion criteria. All criteria utilized should be universally recognized, or reference(s) where detailed explanation can be found should be  given. | 4-5 |
|  |  | 10b. Descriptions of the roles, qualifications and other relevant experience of the researchers (e.g. participant screeners, care providers, outcome assessors, data analysts) in TCM research are recommended. | 6,8,9 |
|  |  | 10c. Descriptions of the qualification and relevant experience of study center(s) involved in  a TCM trial are recommended. | 4 |
| Interventions | *11a.* *Interventionsfor* *each* *group* *with* *sufficient* *detail* *to* *allow* *replication,* *including* *how* *and* *when* *they* *will* *be* *administered* | 11a. 1 Interventions for the experimental group(s) with sufficient detail to allow replication. | 7, Table1 |
|  |  | 11a. 1A. CHM formulae  For fixed CHM formulae  1. Name, source and dosage form (e.g. decoction, granules, powder, pills).  2. Name, source, processing method and dosage of each medical substance. Name of all substances should be presented in at least two types of languages: Chinese (*Pinyin*), Latin or English. Names of the parts of the substances used should be specified.  3. Authentication method of each ingredient, and how, when, where, by whom it will be conducted.  4. Production method of the formula. | 3,7,Table1 |

|  |  | 5. Quality control of each ingredient and the whole formula .  6. Safety assessment of the formula, containing heavy metals and toxic elements test, pesticide residue test, microbial limit test, acute/chronic toxicity test.  7. Dosage of the formula, and how the dosage was determined.  8. Administration route (e.g. oral, external).  For individualized CHM formulae  1. As for fixed CHM formulae, refer to fixed formulae Point 1-8 listed above.  2. Additional information: how, when and by whom the formula will be modified.  For patent proprietary CHM formulae  1. Reference to a publicly available material(s), such as Pharmacopeia, for the details about the composition, dosage, efficacy, safety, and quality control of the formula.  2. Illustration of the details of the formula, namely: i) the proprietary product name (i.e. brand name), ii) name of manufacturer, iii) lot number, iv) production data and expiry date,  v) name and content of added materials, and vi) whether any additional quality control procedures will be conducted.  3. Statement of whether the patent proprietary CHM formula utilized in the study is identical to the publicly available reference. |  |
| --- | --- | --- | --- |
|  |  | 11a. 1B. Acupuncture  1. Treatment environment and participant posture.  2. Number of needle insertions per subject per session (mean and range if possible). | NA |

|  |  | 3. Names and location of acupoints (uni/bilateral). Name of all acupoints should be presented in Chinese (*Pinyin*) and international code.  4. Angle and depth of insertion, which should be presented in a specified unit of measurement or on a particular tissue level.  5. Response sought (e.g. de qi or muscle twitch response).  6. Needle stimulation (e.g. manual, electrical). If electroacupuncture apparatus will be utilized, the brand, manufacturer and frequency should be indicated.  7. Needle retention time.  8. Needle type, including diameter, length, manufacturer and material, etc.  9. Number of treatment sessions.  10. Frequency and duration of treatment sessions. |  |
| --- | --- | --- | --- |
|  |  | 11a. 1C. Moxibustion  1. Treatment environment and participant posture.  2. Number of moxibustion units per subject per session (mean and range if possible).  3. Names and location of acupoints (uni/bilateral). Name of all acupoints should be presented in Chinese (*Pinyin*) and international code.  4. Procedure and technique of moxibustion (e.g. direct/indirect, warming/sparrow-pecking technique, warming needle, moxa box).  5. Response sought (e.g. warm feeling, skin reddening, burning pain).  6. Moxibustion retention time.  7. Materials used for moxibustion (e.g. moxa floss, moxa cone, moxa stick, herbal patches, and their sizes and manufacturers). | NA |

|  |  | 8. Number of treatment sessions.  9. Frequency and duration of treatment sessions. |  |
| --- | --- | --- | --- |
|  |  | 11a.2 Interventions for the control group(s) with sufficient detail to allow replication. | 7, Table1 |
|  |  | 11a.2A. CHM formulae  Placebo control  1. Name and dosage of each ingredient.  2. Description of the similarity of placebo with intervention (e.g. color, smell, taste, appearance, packing).  3. Quality control and safety surveillance, if any.  4. Administration route, dosage and regimen.  5. Production information: when, where, how and by whom the placebo was produced .  Active control  1. If a CHM formula was used, refer to the recommendations of 11a. 1A.  2. If a chemical agent was used, the name, administration route, dosage and regime should be included. | NA |
|  |  | 11a.2B. Acupuncture  Blank/waitlist control  State any special arrangement(s) in pre-treatment, treatment and post-treatment periods corresponding to the experimental intervention (e.g. examinations in pre-treatment period, unaltered lifestyle and medication in treatment period, and compensatory interventions in post-treatment period).  Sham acupuncture or acupuncture-like control  State the comparability of the sham acupuncture or acupuncture-like control and  comprehensively provide details as for the recommendations of 11a. 1B. | NA |

|  |  | 11a.2C. Moxibustion  Blank/waitlist control  State any special arrangement(s) in pre-treatment, treatment and post-treatment periods corresponding to the experimental intervention (e.g. examinations in pre-treatment period, unaltered lifestyle and medication in treatment period, and compensatory interventions in post-treatment period).  Sham moxibustion or moxibustion-like control  State the comparability of the sham moxibustion or moxibustion-like control and  comprehensively provide details as for the recommendations of Intervention 11a. 1C. | NA |
| --- | --- | --- | --- |
|  | *11b.* *Criteria for* *discontinuing* *or* *modifying* *allocated* *interventions for* *a* *given* *trial* *participant* *(e.g,* *drug* *dose* *change* *in* *response* *to* *harms,* *participant* *request,* *or* *improving/worsening* *disease)* |  | 5-7 |
|  | *11c.* *Strategies* *to* *improve* *adherence* *to* *intervention* *protocols,* *and* *any* *procedures for* *monitoring* *adherence* *(e.g,* *drug* *tablet* *return,* *laboratory* *tests)* |  | 8-9 |
|  | *11d.* *Relevant* *concomitant* *care* *and* *interventions* *that* *are* *permitted* *or* *prohibited* *during* *the* *trial* | 11d.2 Descriptions of other interventions that will be administrated to experimental and/or control groups are recommended (e.g. rescue interventions), with enough details to allow replication. | 7 |
| Outcomes | *12.* *Primary,* *secondary,* *and* *other* *outcomes,* *including* *the* *specific* *measurement* *variable* *(eg,* *systolic* *blood* *pressure),* *analysis* *metric* *(eg,* *change from* *baseline,final value,* *time* *to* *event), method* *of* | 12a. Provide the rationale of TCM-related indexes as outcomes (e.g. the change of degree  and scope of symptoms and signs related to *Pattern* differentiation). | 8 |

|  | *aggregation* *(e.g,* *median,* *proportion),* *and* *time* *point for* *each* *outcome.* *Explanation* *of* *the* *clinical* *relevance* *of* *chosen* *efficacy* *and* *harm* *outcomes* *is* *strongly* *recommended* | 12b. Provide the details of the TCM-related outcomes assessment, including i) the  measuring methods and standard (e.g. frequency, severity rating scale of symptoms and signs, verified *Pattern* questionnaire, time points for assessment and corresponding rationale), ii) assessor qualification (e.g. relevant assessment experience, years in clinical practice), iii) methods used to enhance the quality of assessment (e.g. multiple repeated observation, training of assessors), and iv) related reference(s). | 8 |
| --- | --- | --- | --- |
| Participant  timeline | *13.* *Time* *schedule* *of* *enrolment,* *interventions* *(including* *any* *run-ins* *and* *washouts),* *assessments,* *and* *visits for* *participants.* *A* *schematic* *diagram* *is* *highly* *recommended* |  | 4,7,  Figure 1,  Table 2 |
| Sample size | *14.* *Estimated* *number* *of* *participants* *needed* *to* *achieve* *study* *objectives* *and* *how* *it* *was* *determined,* *including* *clinical* *and* *statistical* *assumptions* *supporting* *any* *sample* *size* *calculations* |  | 6 |
| Recruitment | *15.* *Strategies for* *achieving* *adequate* *participant* *enrolment* *to* *reach* *target* *sample* *size* |  | 3-4 |
| ***Assignment*** ***of*** ***interventions*** ***(for*** ***controlled*** ***trials)*** | | |  |
| Sequence  generation | *16a.* *Method* *of* *generating* *the* *allocation* *sequence* *(e.g, computer-generated* *random* *numbers)* *and* *list* *of* *any* *factors for* *stratification.To* *reduce* *predictability* *of* *a* *random* *sequence,* *details* *of* *any* *planned* *restriction* *(e.g,* *blocking)* *should* *be* *provided* *in* *a* *separate* *document* *that is* *unavailable* *to* *those* *who* *enroll* *participants* *or* *assign interventions* |  | 6-7 |
| Allocation  Concealment  mechanism | *16b.* *Mechanism* *of* *implementing* *the* *allocation* *sequence* *(e.g, central* *telephone;* *sequentially* *numbered,* *opaque,sealed* *envelopes) describing* *any* *steps* *to* *conceal* *the* *sequence* *until* *interventions* *are* *assigned* |  | 6-7 |

| Implementation | *16c.* *Who* *will* *generate* *the* *allocation* *sequence,* *who* *will* *enroll* *participants,* *and* *who* *will* *assign* *participants* *to* *interventions* |  | 6-7 |
| --- | --- | --- | --- |
| Blinding  (masking) | *17a.* *Who* *will* *be* *blinded* *after* *assignment* *to* *interventions* *(e.g,* *trial* *participants,* *care* *providers,* *outcome* *assessors,* *data* *analysts)* *and* *how* |  | 6-7 |
|  | *17b.* *If* *blinded,* *circumstances* *under* *which* *unblinding* *is* *permissible* *and* *procedure for* *revealing* *a* *participant’s* *allocated* *intervention* *during* *the* *trial* |  | 6-7 |
| ***Data*** ***collection,*** ***management,*** ***and*** ***analysis*** | | |  |
| Data collection methods | *18a.* *Plans for* *assessment* *and* *collection* *of* *outcome,baseline,* *and* *other* *trial* *data,* *including* *any* *related* *processes* *to* *promote* *data* *quality* *(e.g,* *duplicate* *measurements,* *training* *of* *assessors)* *and* *a* *description* *of* *study* *instruments* *(e.g,* *questionnaires,* *laboratory* *tests) along* *with* *their* *reliability* *and* *validity,* *if* *known.* *Reference* *to* *where* *data* *collection forms* *can* *be found,* *if* *not* *in* *the* *protocol* | 18a. When trial targeting on TCM *Pattern*, or a WM-defined disease with a specific TCM *Pattern*, baseline data about TCM *Pattern* should be provided. | 8-9 |
|  | *18b.* *Plans* *to* *promote* *participant* *retention* *and* *complete* *follow-up,* *including* *list* *of* *any* *outcome* *data* *to* *be* *collected for* *participants* *who* *discontinue* *or* *deviate from intervention* *protocols* |  | 8-9 |

| Data  management | *19.* *Plans for* *data* *entry,* *coding,* *security,* *and* *storage, including* *any* *related* *processes* *to* *promote* *data* *quality* *(e.g,* *double* *data* *entry;* *range* *checks for* *data* *values).* *Reference* *to* *where* *details* *of* *data* *management* *procedures* *can* *be found,* *if* *not* *in* *the* *protocol* |  | 9 |
| --- | --- | --- | --- |
| Statistical  methods | *20a.* *Statistical* *methods for* *analysing* *primary* *and* *secondary* *outcomes.* *Reference* *to* *where* *other* *details* *of* *the* *statistical* *analysis* *plan* *can* *be found,* *if* *not* *in* *the* *protocol* |  | 9-10 |
|  | *20b.* *Methods for* *any* *additional* *analyses* *(e.g,* *subgroup* *and* *adjusted* *analyses)* |  | 9-10 |
|  | *20c.* *Definition* *of* *analysis* *population* *relating* *to* *protocol non-adherence* *(e.g,* *as* *randomised* *analysis),* *and* *any* *statistical* *methods* *to* *handle* *missing* *data* *(e.g,* *multiple* *imputation)* |  | 9-10 |
| ***Monitoring*** | | |  |
| Data  monitoring | *21a.* *Composition* *of* *data* *monitoring* *committee* *(DMC);* *summary* *of* *its* *role* *and* *reporting* *structure;* *statement* *of* *whether* *it* *is* *independent from* *the* *sponsor* *and* *competing* *interests;* *and* *reference* *to* *where further* *details* *about* *its* *charter* *can* *be found,* *if* *not* *in* *the* *protocol.* *Alternatively,* *an* *explanation* *of* *why* *a* *DMC* *is* *not* *needed* |  | 9 |
|  | *21b.* *Description* *of* *any* *interim* *analyses* *and* *stopping* *guidelines,* *including* *who* *will* *have* *access* *to* *these* *interim* *results* *and* *make* *the final* *decision* *to* *terminate* *the* *trial* |  | 9 |

| Harms | *22.* *Plans for* *collecting,* *assessing,* *reporting,* *and managing* *solicited* *and* *spontaneously* *reported* *adverse* *events* *and* *other* *unintended* *effects* *of* *trial* *interventions* *or* *trial* *conduct* |  | 8-9 |
| --- | --- | --- | --- |
| Auditing | *23.* *Frequency* *and* *procedures for* *auditing* *trial* *conduct,* *if* *any,* *and* *whether* *the* *process* *will* *be* *independent from* *investigators* *and* *the* *sponsor* |  | 9 |
| ***Ethics*** ***and*** ***dissemination*** | | |  |
| Research ethics  approval | 1. *Plans for* *seeking* *research* *ethics committee/institutional* *review*   *board* *(REC/IRB)* *approval* |  | 12 |
| Protocol  amendments | *25.* *Plans for* *communicating* *important* *protocol* *modifications* *(e.g,* *changes* *to* *eligibility* *criteria,* *outcomes,* *analyses)* *to* *relevant* *parties* *(e.g,* *investigators,* *REC/IRBs,* *trial* *participants,* *trial registries, journals,* *regulators)* |  | 12 |
| Consent or  assent | *26a.* *Who* *will* *obtain* *informed* *consent* *or* *assent from potential* *trial* *participants* *or* *authorised* *surrogates,and* *how* *(see* *Item* *32)* |  | 4,12 |
|  | *26b.* *Additional* *consent* *provisionsfor* *collection* *and* *use* *of* *participant* *data* *and* *biological* *specimens* *in* *ancillary* *studies,* *if* *applicable* |  | NA |
| Confidentiality | *27.* *How* *personal* *information* *about* *potential* *and* *enrolled* *participants* *will* *be* *collected,* *shared,* *and* *maintained* *in* *order* *to* *protect* *confidentiality* *before,* *during,* *and* *after the* *trial* |  | 12 |
| Declaration of interests | *28.* *Financial* *and* *other* *competing* *interests for* *principal* *investigators for* *the* *overall* *trial* *and* *each* *study* *site* |  | 16 |

| Access to data | *29.* *Statement* *of* *who* *will* *have* *access* *to* *the final* *trial* *data set,* *and* *disclosure* *of* *contractual* *agreements* *that* *limit* *such* *access for* *investigators* |  | 9, 12 |
| --- | --- | --- | --- |
| Ancillary and post-trial care | *30.* *Provisions,* *if* *any,for* *ancillary* *and* *post-trial* *care,* *and* *for* *compensation* *to* *those* *who* *suffer* *harm from* *trial* *participation* |  | 8 |
| Dissemination policy | *31a.* *Plans for* *investigators* *and* *sponsor* *to* *communicate* *trial* *results* *to* *participants,* *healthcare* *professionals,* *the* *public,* *and* *other* *relevant* *groups* *(e.g,* *via* *publication,* *reporting* *in* *results* *databases,* *or* *other* *data* *sharing* *arrangements),* *including* *any* *publication* *restrictions* | Plan for raw data sharing, if any. The contents should contain: i) when the data will become available; ii) how the data will be shared iii) what data in particular will be shared; iv) who could acquire the data; v) through what access data will be shared. | 12 |
|  | *31b.* *Authorship* *eligibility* *guidelines* *and* *any* *intended* *use* *of* *professional* *writers* |  | 12 |
|  | *31c.* *Plans,* *if* *any,for* *granting* *public* *access* *to* *the full* *protocol,* *participant* *level* *data set,* *and* *statistical* *code* |  | NA |
| ***Appendices*** | | |  |
| Informed  consent  materials | *32.* *Model* *consent form* *and* *other* *related* *documentation* *given* *to* *participants* *and* *authorised* *surrogates* |  | NA |
| Biological  specimens | *33.* *Plans for* *collection,* *laboratory* *evaluation,* *and* *storage* *of* *biological* *specimens for* *genetic* *or* *molecular* *analysis* *in* *the* *current* *trial* *and for future* *use* *in* *ancillary* *studies,* *if* *applicable* |  | NA |

Abbreviations: CHM, Chinese herbal medicine; SPIRIT, Standard Protocol Items: Recommendations for Interventional Trials; TCM, traditional Chinese medicine; WM,

Western medicine
